# Androgen receptor dampens tissue factor expression via nuclear factor‐κB and early growth response protein 1

**DOI:** 10.1111/jth.13971

**Published:** 2018-03-13

**Authors:** B. Hoesel, M. Mussbacher, B. Dikorman, M. Salzmann, A. Assinger, L. Hell, J. Thaler, J. Basílio, B. Moser, U. Resch, H. Paar, N. Mackman, J. A. Schmid

**Affiliations:** ^1^ Institute of Vascular Biology and Thrombosis Research Center for Physiology and Pharmacology Medical University of Vienna Vienna Austria; ^2^ Department of Medicine I Clinical Division of Hematology and Hemostaseology Medical University of Vienna Vienna Austria; ^3^ University of North Carolina at Chapel Hill Chapel Hill NC USA

**Keywords:** androgen receptor, early growth response protein 1, NF‐κB, prostate cancer, tissue factor, venous thromboembolism

## Abstract

**Essentials**

- Androgen deprivation increases the rate of venous thromboembolism in prostate cancer patients.
- We characterized androgen receptor‐mediated tissue factor regulation in prostate epithelial cells.
- Androgen receptor is dampening tissue factor expression in prostate epithelial cells.
- Androgen deprivation could enhance tissue factor expression and raise venous thromboembolism rates.

**Summary:**

**Background**

Prostate cancer is one of the leading causes of cancer death in men. Advanced prostate cancer is usually treated by androgen deprivation therapy (ADT), which is aimed at reducing circulating testosterone levels to reduce cancer growth. There is growing evidence that ADT can increase the rate of venous thromboembolism (VTE) in prostate cancer patients. The tissue factor (TF) gene is one of the most important mediators of coagulation and VTE, but, so far, there are limited data on androgen receptor (AR)‐mediated TF gene expression.

**Objectives**

To characterize AR‐mediated TF regulation *in vitro* and *in vivo*.

**Methods**

We used the androgen‐dependent prostate cancer cell lines LNCaP and MyC‐CaP to test whether TF expression is regulated by AR. Furthermore, we cloned the TF gene promoter into a luciferase reporter vector to identify the transcription factor‐binding sites that mediate TF regulation downstream of AR. Finally, we used castration experiments in mice to characterize AR‐mediated TF regulation *in vivo*.

**Results**

TF is directly regulated by AR. In LNCaP cells, nuclear factor‐κB signaling and EGR1 mediate TF expression. By using castration experiments in mice, we could detect upregulation of TF and early growth response protein 1 mRNA and protein expression in prostate epithelial cells.

**Conclusion**

AR is crucial for dampening TF expression, which could be important for increased TF expression and TF‐positive microvesicle release in androgen‐deprived prostate cancer patients.

## Introduction

In 2018, ∼ 164 690 new cases of prostate cancer will be diagnosed in the USA, which makes it the second most diagnosed cancer after lung cancer 1. Primary prostate cancer is usually treated with surgery or radiation therapy 2. In cases of biochemical recurrence, patients are typically treated with androgen deprivation therapy (ADT), which is aimed at reducing the levels of male hormones to reduce cancer growth 3.

Prostate cancer is a known risk factor for venous thromboembolism (VTE), which includes deep vein thrombosis and pulmonary embolism 4, 5. There is growing evidence that ADT increases the rate of VTE in prostate cancer patients, suggesting that low levels of testosterone can induce a hypercoagulable state 6, 7.

One of the most important mediators of coagulation is tissue factor (TF). TF is a transmembrane glycoprotein that has important functions in blood clotting and hemostasis, but also in tumor progression and metastasis 8. Cancer cells can release TF‐positive microvesicles (MVs) into the bloodstream, which may contribute to blood clotting and the development of VTE 9, 10. In addition, it has been reported that high TF expression levels in prostate cancer significantly correlate with Gleason score and lethality, and promote angiogenesis 11, 12, 13, 14.

In prostate tumors, TF expression appears to be confined to malignant luminal epithelial cells 13. In epithelial cells, TF expression is regulated by many stimuli, including growth factors, such as vascular endothelial growth factor, or inflammatory mediators, such as lipopolysaccharide, tumor necrosis factor‐α, and interleukin‐1β. Regulation of TF gene expression is mediated by a well‐defined set of transcription factor‐binding sites in the TF gene promoter, including sites binding activator protein 1 (AP‐1), nuclear factor‐κB (NF‐κB), specificity protein 1 (SP1), and early growth response protein 1 (EGR1) 15.

Despite a potential role for TF in prostate cancer, an analysis of androgen receptor (AR)‐mediated regulation of TF expression has so far not been reported. We show that TF expression and activity are regulated after dihydrotestosterone (DHT) treatment in the androgen‐dependent prostate cancer cell lines LNCaP and MyC‐CaP. This DHT‐mediated TF regulation is AR‐dependent, as it could be blocked by addition of the AR antagonist bicalutamide. Furthermore, we cloned the TF gene promoter into a luciferase reporter vector and analyzed the contribution of known transcription factor‐binding sites to AR‐mediated TF regulation. Using this approach, we found that TF regulation is mediated through NF‐κB and EGR1 transcription factor‐binding sites in LNCaP cells. Finally, by using castration experiments in mice, we provide evidence that an AR–NF‐κB–EGR1 signaling axis is also operative in prostate epithelial cells *in vivo*.

## Materials and methods

### Cell lines and cell culture

LNCaP and MyC‐CaP cells were purchased from ATTC. PC3 and DU145 cell lines were a kind gift from M. Unseld (Medical University Vienna; Comprehensive Cancer Center). LNCaP, PC3 and DU145 cells were routinely maintained in normal RPMI medium containing 10% fetal bovine serum (FBS), phenol red, and antibiotics. MyC‐CaP cells were routinely maintained in normal Dulbecco's modified Eagle's medium (DMEM). For DHT stimulation experiments, LNCaP, MyC‐CaPs, PC3 and DU145 cells were seeded at 50–60% density in RPMI medium or DMEM, without phenol red, containing 5% charcoal‐stripped FBS (Sigma, Vienna, Austria). Transient transfections of all reporter and expression plasmids were performed with Turbofec (Thermo Fisher Scientific, Vienna, Austria), according to the manufacturer's instructions, on the second day of starvation. After 3 days of starvation, cells were stimulated with the indicated DHT concentrations and analyzed at the indicated time points. DHT and bicalutamide were purchased from Sigma.

### Mice

Male C57Bl6/J mice aged 6–8 weeks were purchased from Charles River (Sulzfeld, Germany). At 8–10 weeks mice were either castrated or sham‐operated. Two weeks after surgery, mice were injected subcutaneously with Miglyol 812 (control) or with DHT (20 μg g^−1^ mouse) dissolved in Miglyol 812, for three consecutive days. Finally, mice were killed, prostates were harvested, and RNA was extracted with the peqGOLD Total RNA Kit (VWR, Vienna, Austria) according to the manufacturer's protocol. *In vivo* experiments complied with institutional guidelines for animal experimentation; ethical approval was obtained from the Federal Ministry for Science and Research, Vienna, Austria (BMWFW‐66.009/0332‐WF/V/3b/2015).

### Plasmids and cloning

p2106‐TF and p278‐TF fragments were derived from previously published plasmids 16, released by the use of *Hin*dIII or *Kpn*I, respectively, and subsequently ligated in the pNL1.1 Nanoluc Reporter vector (Promega, Mannheim, Germany). Serial deletions of the p278‐TF promoter were created with specific primers, as summarized in Table S1, and the NEB Q5 Site‐Directed Mutagenesis Kit (New England Biolabs, Frankfurt, Germany), according to the manufacturer's protocol. The NF‐κB reporter luciferase (5× NF‐κB; Stratagene, Santa Clara, CA, USA) has been previously described 17. The pcDNA3/HA‐mAR expression plasmid was a kind gift from the laboratory of N. M. Greenberg 18, and was subcloned into the DSRed‐C1 (Clontech, Saint‐Germain‐en‐Laye, France) vector after *Xho*I and *Bam*HI digestion. The EGR1 Nanoluc reporter was created by digesting an EGR1 luciferase reporter (pGL4[luc2P/hEGR1/Hygro]; Promega) with *Acc*I and *Hin*dIII. The released fragment was subsequently cloned into the pNL1.1 Nanoluc Reporter vector (Promega). Plasmids were checked by sequencing before use.

### Luciferase assays

Cells were transiently transfected with the TF gene promoter or NF‐κB and EGR1 reporter constructs, and a vector constitutively expressing β‐galactosidase for normalization purposes (driven by a ubiquitin promoter: PUB6/V5‐His/LacZ from Thermo Fisher Scientific). Cells were lysed in passive lysis buffer (0.1 m KH_2_PO_4_, 0.1% Triton X‐100). Nanoluc luminescence was measured as recommended by the manufacturer (Promega). Firefly luminescence and β‐galactosidase were measured as previously described 19.

### Quantitative PCR (qPCR)

qPCR was performed with SYBR Green reagents (Thermo Fisher Scientific) as previously described 19. The primers used for qPCR analysis are summarized in Table S1. For correlation analysis, we calculated 2^−ΔΔCT^ values of the respective target genes in DU145, PC3 and LNCAP cells. Glyceraldehyde‐3‐phosphate dehydrogenase (GAPDH) was used as a housekeeping gene. Mean PCR efficiency was calculated with linreg 20. 2^−ΔΔCT^ values were used for calculation of the Pearson *r* coefficient (relative to TF expression), and statistical analysis was performed with graphpad prism 7.0 software.

### Avidin–biotin complex DNA (ABCD) assay

The ABCD assay was performed as previously described 19, 21. The oligonucleotides used in this study were: NF‐κB_for, Bio‐GGGAAATTCCCTTGGAAATTCCCTTGGAAATT‐CCCCTTGGAAATTCC; and NF‐κB_rev, Bio‐GGAATTTCCAAGGGGAATTTCCAAGGG‐AATTTCCAAGGGAATTTCCC.

### Western blotting and immunohistochemistry

Western blotting was performed according to standard procedures. The antibodies used were: anti‐p65 (Santa Cruz, Heidelberg, Germany: sc‐109), anti‐p50 (Cell Signaling, Frankfurt, Germany: #3035), anti‐β‐tubulin (Santa Cruz: sc‐9104), anti‐AR (Merck, Vienna, Austria: 06‐680), anti‐TF (Abcam, Cambridge, UK: AB151748), anti‐IκBα (Santa Cruz: sc‐371), anti‐c‐Rel (Cell Signaling: #4727), anti‐EGR1 (Santa Cruz: sc‐110), anti‐SP1 (Cell Signaling: #9389), and anti‐GAPDH (Novus Biologicals, Littleton, CO, USA: NBP1‐47339). Immunohistochemistry was performed with a Vectastain Elite ABC horseradish peroxidase (HRP) Kit (Vectorlabs, Burlingame, CA, USA) according to the manufacturer's protocol. Antigen retrieval was performed by boiling slides for 20 min in 10 mm sodium citrate buffer (pH 6). HRP was developed with a Vectorlabs 3,3′‐diaminobenzidine peroxidase (HRP) Substrate Kit according to the manufacturer's protocol. Slides were counterstained with hematoxylin. The antibodies used for immunohistochemistry were anti‐TF (Abcam: AB151748) and anti‐EGR1 (Cell Signaling: #4154).

### TF activity assay

TF activity was determined essentially as previously described 22. As DHT treatment induces proliferation of LNCaP and MyC‐CaP cells, we normalized TF activity in the cell culture supernatant to the total protein content of attached cells. The total protein content was determined with a Pierce BCA Protein Assay Kit (Thermo Fisher Scientific) according to the manufacturer's protocol.

### Flow cytometry

LNCaP cells were stimulated with different concentrations of DHT (1–100 nm) for 48 h, harvested by scraping, and finally fixed in 1% paraformaldehyde. To determine the total cellular TF protein content in LNCaPs, cells were permeabilized with 0.1% Triton X‐100 in phosphate‐buffered saline (PBS) for 15 min, and labeled with anti‐CD142–fluorescein isothiocyanate (anti‐TF) (CLB/TF5; Cat. No. MA1‐82810; Thermo Fisher Scientific). Mean fluorescence intensity (MFI) was analyzed with a BD Accurri C6 flow cytometer and BD Accuri C6 Samples software (Becton Dickinson, Schwechart, Austria). MyC‐CaP cells were stimulated with different concentrations of DHT (1–100 nm) for 48 h, and detached with Versene solution at 4 °C (0.5 mm EDTA in PBS). To determine the surface TF protein content, MyC‐CaP cells were labeled with anti‐TF–phycoerythrin (R&D Systems, Minneapolis, MN, USA: Fab3178P). Live cells were separated by staining with SYTOX AADvanced Dead Cell stain (Thermo Fisher Scientific). MFI was analyzed with a Cytoflex S cytometer and Cytexpert software 2.0 (Beckman Coulter, Vienna, Austria).

### Gene set enrichment analysis (GSEA)

GSEA was performed as previously described 23. In brief, GSEA is a computational method that determines whether a defined set of genes show a significant difference between two biological states. For our analysis, the probe set IDs, which annotate to the respective genes to be analyzed (those encoding TF, EGR1, SP1, p65, p50, p52, IκBα, SP1, and c‐Rel), were set as phenotype. GSEA software then calculated whether the expression profile of these genes was enriched within an AR‐induced or AR‐repressed gene set. The Pearson correlation coefficient was used as the ranking metric. For AR‐induced or AR‐repressed genes, we used a previously published list of genes 24. The dataset used for analysis was GSE21032 25.

### Statistics

Statistical analysis was performed with graphpad prism 7.0. Data were analyzed with one‐way anova and Dunett's multiple comparison test for groups larger than two, or Student's *t*‐test.

## Results

### TF expression is regulated by DHT in LNCaP and MyC‐CaP cells

**Figure 1:**
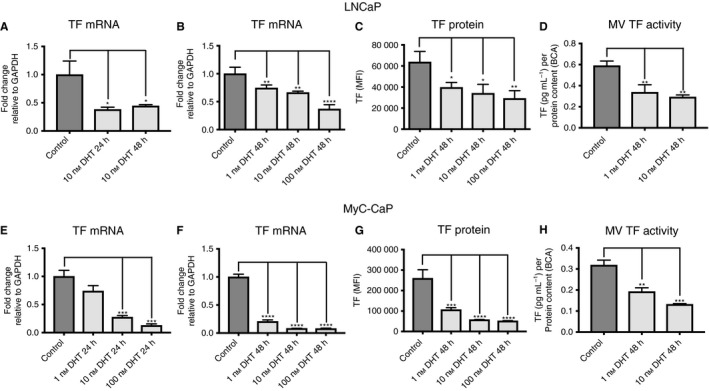
Tissue factor (TF) expression is dihydrotestosterone (DHT)‐dependent in LNCaP cells and MyC‐CaP cells. (A) Quantitative PCR (qPCR) for TF mRNA in control LNCaP cells and after 24 h and 48 h of 10 nm DHT treatment (*n* = 6). (B) qPCR for TF mRNA in control LNCaP cells and after 48 h of 1, 10 and 100 nm DHT treatment (*n* = 6). (C) Fluorescence‐activated cell sorting (FACS) analysis for TF protein in control LNCaP cells and after 48 h of 1, 10 and 100 nm DHT treatment (*n* = 6). (D) TF activity of microvesicles (MVs) isolated from cell culture supernatants in control LNCaP cells and after 48 h of 1 nm and 10 nm DHT treatment (*n* = 9). (E) qPCR for TF mRNA in control MyC‐CaP cells and after 24 h of 1, 10 and 100 nm DHT treatment (*n* = 6). (F) qPCR for TF mRNA in control MyC‐CaP cells and after 48 h of 1, 10 and 100 nm DHT treatment (*n* = 6). (G) FACS analysis for TF protein in control MyC‐CaP cells and after 48 h of 1, 10 and 100 nm DHT treatment (*n* = 3). (H) TF activity of MVs isolated from cell culture supernatants in control MyC‐CaP cells and after 48 h of 1 nm and 10 nm DHT treatment (*n* = 6). *P ≤ 0.05, **P ≤ 0.01, ***P ≤ 0.001, ****P ≤ 0.0001. Error bars represent standard error of the mean. GAPDH, glyceraldehyde‐3‐phosphate dehydrogenase; MFI, mean fluorescence intensity.

To test for a potential connection between TF and AR, we first stimulated LNCaP cells in a time‐dependent and concentration‐dependent manner with DHT. First, we observed that TF mRNA was significantly downregulated after 24 h and 48 h of 10 nm DHT treatment (Fig. 1A). Moreover, DHT‐induced TF downregulation was concentration‐dependent after 48 h of DHT treatment (Fig. 1B). In addition, we found concentration‐dependent downregulation of TF protein in LNCaPs, as determined by fluorescence‐activated cell sorting analysis (Fig. 1C). Finally, we observed DHT‐dependent downregulation of TF activity in cell culture supernatants of LNCaP cells (Fig. 1D). As a next step, we stimulated MyC‐CaP cells in a time‐dependent and concentration‐dependent manner with DHT. In line with previous results, TF mRNA was significantly downregulated after 24 h and 48 h of DHT treatment (Fig. 1E,F). Furthermore, we observed DHT‐dependent downregulation of TF activity in cell culture supernatants of MyC‐CaP cells (Fig. 1H). In summary, these findings indicate that TF mRNA, protein and activity are downregulated by DHT treatment in LNCaP and MyC‐CaP cells.

### Basal TF regulation in prostate cancer epithelial cells

**Figure 2:**
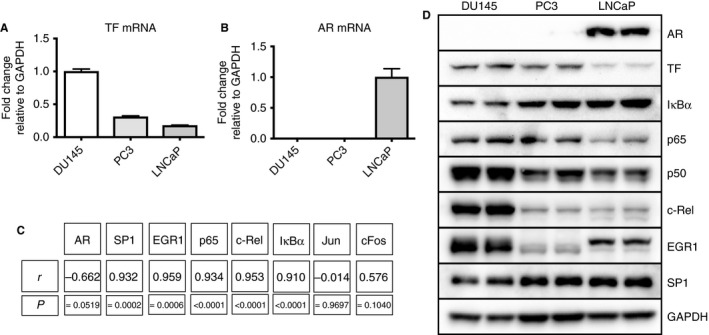
Basal tissue factor (TF) and androgen receptor (AR) mRNA expression in prostate cancer cell lines. (A) Basal TF mRNA expression levels in DU145, PC3 and LNCaP cells as determined by quantitative PCR (qPCR) (*n* = 6). (B) Basal AR mRNA expression levels in DU145, PC3 and LNCaP cells as determined by qPCR (*n* = 6). (C) Correlation analysis of basal TF mRNA expression levels with basal mRNA expression levels of AR, specificity protein 1 (SP1), early growth response protein 1 (EGR1), p65, c‐Rel, IκBα, Jun and cFos mRNA (*n* = 6). (D) Western blot analysis of AR, TF, IκBα, p65, p50, c‐Rel, EGR1, SP1 and glyceraldehyde‐3‐phosphate dehydrogenase (GAPDH) protein expression in DU145, PC3 and LNCaP cells. Error bars represent standard error of the mean.

To better understand TF expression and regulation in prostate cancer epithelial cells, we first determined basal TF mRNA levels in DU145, PC3 and LNCaP cells by using qPCR (Fig. 2A). In general, we observed the highest TF mRNA expression levels in DU145 cells. Furthermore, we measured AR, SP1, Jun, cFos, EGR1, IκBα, p65 and c‐Rel mRNA expression levels by qPCR, and correlated their expression profile with TF expression. Here, we found that the expression levels of SP1, EGR1 and all NF‐κB signaling components tested correlated with TF expression (Fig. 2C). We did not detect any significant correlation of the TF mRNA expression level with Jun and cFos expression levels. As a next step, we performed western blot analysis to determine whether IκBα, p65, p50, c‐Rel, EGR1 and SP1 protein levels also correlate with TF expression. In line with previous results, we detected high TF protein expression in DU145 cells and low TF protein expression in LNCaP cells (Fig. 2D). Furthermore, we confirmed a correlation between the expression profile of TF and the expression of NF‐κB signaling components. In particular, we detected an expression profile of the transcription factors p65, p50 and c‐Rel that was reminiscent of TF protein expression in these cells (Fig. 2D). SP1 and EGR1 protein expression, in contrast, did not correlate with TF protein expression. In summary, these data suggest that basal TF expression might be mediated by the NF‐κB signaling pathway in prostate cancer epithelial cells.

### TF regulation is dependent on AR

**Figure 3:**
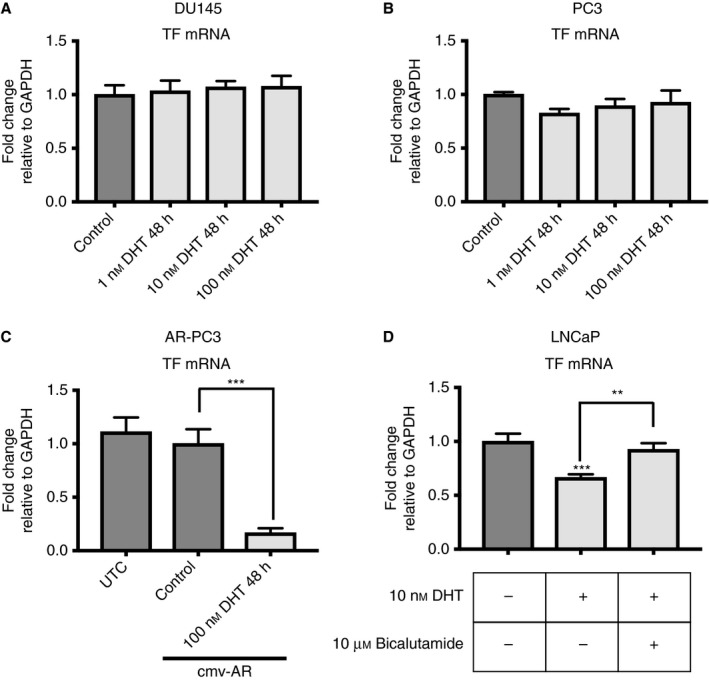
Tissue factor (TF) expression is androgen receptor (AR)‐dependent. (A) Quantitative PCR (qPCR) for TF mRNA in control DU145 cells and after 48 h of 1, 10 and 100 nm dihydrotestosterone (DHT) treatment (*n* = 6). (B) qPCR for TF mRNA in control PC3 cells and after 48 h of 1, 10 and 100 nm DHT treatment (*n* = 6). (C) qPCR for TF mRNA in untransfected control (UTC) and AR‐transfected control PC3 cells and after 48 h of 100 nm DHT treatment (*n* = 6). (D) qPCR for TF mRNA in control LNCaP cells and LNCaP cells treated for 48 h with 10 nm DHT; 10 μm bicalutamide was added as indicated (*n* = 6). **P ≤ 0.01, ***P ≤ 0.001. Error bars represent standard error of the mean. cmv‐AR, plasmid containing a cmv promoter followed by an AR cDNA; GAPDH, glyceraldehyde‐3‐phosphate dehydrogenase.

DHT mediates its effects not only through AR but also through other receptors, including the glucocorticoid receptor 26. To address whether the DHT‐mediated changes in TF gene expression are indeed mediated through AR, we first stimulated DU145 and PC3 cells with DHT in a concentration‐dependent manner, as these cells do not express AR at a significant level (Fig. 2B,D). DHT treatment did not induce any significant changes in TF mRNA expression in these cell lines (Fig. 3A,B). Next, we transiently transfected PC3 cells with an AR expression plasmid, and then treated them with 100 nm DHT for 48 h. We detected significant downregulation of TF mRNA expression (Fig. 3C). Finally, we inhibited DHT‐mediated AR signaling by adding 10 μm bicalutamide to LNCaP cells. In line with a direct AR–TF signaling axis, bicalutamide blocked DHT‐mediated downregulation of TF expression in LNCaP cells (Fig. 3D). These findings indicate that TF expression is directly regulated by AR.

### AR‐mediated regulation of TF expression in LNCaP cells

**Figure 4:**
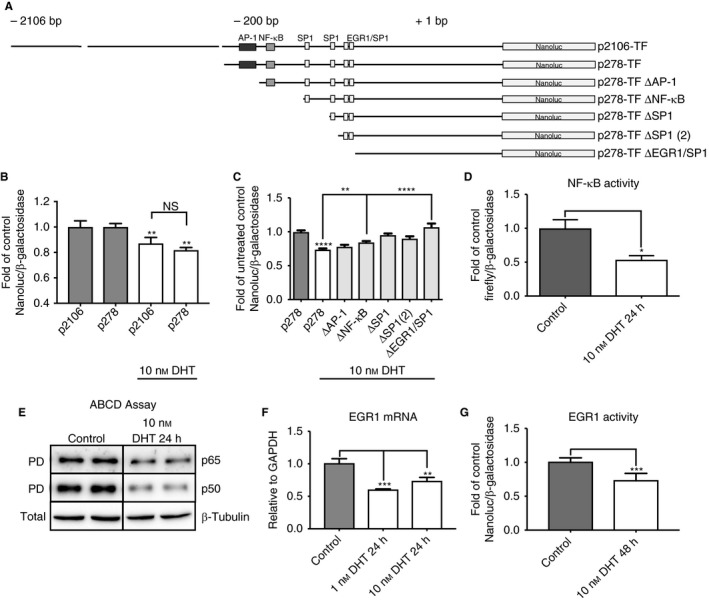
Androgen receptor‐mediated tissue factor (TF) regulation in LNCaP cells. (A) Nanoluc reporter plasmids used in this study. (B) Comparison of the dihydrotestosterone (DHT)‐mediated regulation of 2106‐bp and 278‐bp TF reporter vectors in LNCaP cells (*n* = 12) (C) DHT‐mediated downregulation of a TF reporter vector construct in LNCaP cells is dependent on the binding sites for nuclear factor‐κB (NF‐κB) and early growth response protein 1 (EGR1) in the TF gene promoter (*n* = 10). To account for basal expression changes resulting from the deletion of the respective transcription factor‐binding sites, we normalized the DHT‐treated conditions to their respective untreated controls (except for the 278‐bp control plasmid, which is depicted as control and DHT‐treated). Every other DHT‐treated condition is depicted as fold of its relative untreated control. (D) NF‐κB activity after 24 h of 10 nm DHT treatment in LNCaP cells as determined by luciferase assay (*n* = 6). (E) p65 and p50 DNA‐binding activity in control LNCaP cells and after treatment for 24 h with 10 nm DHT, as determined by avidin–biotin complex DNA (ABCD) assay. (F) EGR1 mRNA expression as determined by qPCR in control LNCaP cells and after treatment for 24 h with 10 nm DHT (*n* = 6). (G) EGR1 activity as determined by luciferase assay in control LNCaP cells and after treatment for 48 h with 10 nm DHT (*n* = 6). *P ≤ 0.05, **P ≤ 0.01, ***P ≤ 0.001, ****P ≤ 0.0001. Error bars represent standard error of the mean. AP‐1, activator protein 1; GAPDH, glyceraldehyde‐3‐phosphate dehydrogenase; NS, not significant; PD, pulldown; SP1, specificity protein 1.

To better understand DHT‐mediated regulation of TF expression in LNCaP cells, we cloned 2106‐bp and 278‐bp fragments of the TF gene promoter into a luciferase reporter vector (Fig. 4A). The 278‐bp fraction of the TF gene promoter was previously shown to contain the most important elements for TF regulation in epithelial cells 15. In line with these results, we did not detect any significant differences in normalized Nanoluc activity after 48 h of 10 nm DHT treatment when we used a 2106‐bp or 278‐bp TF gene promoter fragment in LNCaP cells (Fig. 4B). Next, we serially deleted the binding sites for AP‐1, NF‐κB, SP1 and EGR1 from the p278‐TF plasmid, and determined normalized Nanoluc activity for these constructs in DHT‐treated LNCaP cells (Fig. 4C). In general, we observed that the first and second SP1‐binding sites did not have a role in the DHT‐induced downregulation of Nanoluc activity in LNCaP cells. In contrast, significant changes were detected after abrogation of the binding sites for NF‐κB and EGR1. We detected significant enhancement of the DHT‐reduced Nanoluc activity after deletion of the NF‐κB‐binding site, and this was further enhanced after deletion of the EGR1‐binding site (Fig. 4C). We thus analyzed NF‐κB and EGR1 signaling events in control and DHT‐treated LNCaP cells in more detail. To determine DHT‐mediated effects on NF‐κB activity in LNCaP cells, we determined the luciferase activity of a reporter plasmid bearing consensus NF‐κB‐binding sites (Fig. 4D). We also determined p65‐binding and p50‐binding activity by using ABCD assays (Fig. 4E). In accordance with previously published data 27, 28, we found that both luciferase and p50‐binding and p65‐binding activities were reduced after 24 h of 10 nm DHT treatment in LNCaP cells.

To assess EGR1‐mediated TF regulation in LNCaP cells, we determined EGR1 mRNA expression levels and EGR1 activity by using a reporter plasmid. In line with a regulatory function for EGR1, DHT induced downregulation of EGR1 mRNA expression after 24 h of DHT treatment (Fig. 4F). Furthermore, we detected significantly reduced EGR1 activity after 48 h of 10 nm DHT treatment (Fig. 4G). In summary, these data indicate that DHT downregulates TF expression in LNCaP cells by repressing NF‐κB‐mediated signaling and by reducing EGR1 expression.

### TF expression is upregulated after castration *in vivo*

**Figure 5:**
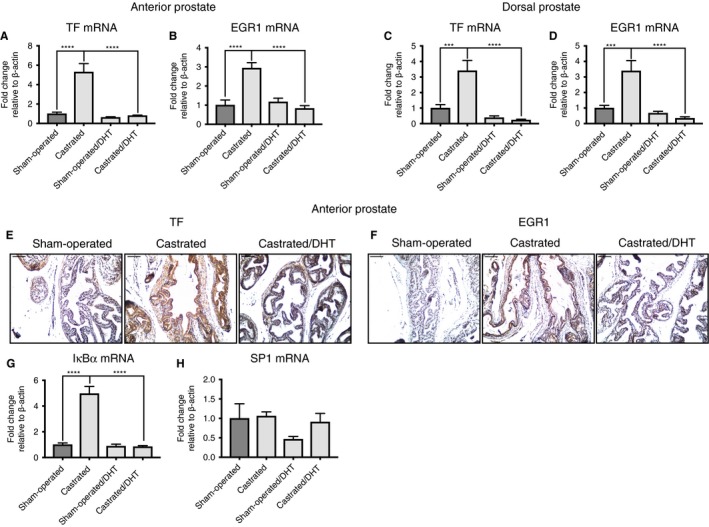
Castration enhances tissue factor (TF) protein expression in prostate epithelial cells. (A, B) Quantitative PCR (qPCR) for TF and early growth response protein 1 (EGR1) mRNA in anterior prostates of sham‐operated, castrated, sham‐operated dihydrotestosterone (DHT)‐treated and castrated DHT‐treated mice (*n* = 10). (C, D) qPCR for TF and EGR1 mRNA in dorsal prostates of sham‐operated, castrated, sham‐operated DHT‐treated and castrated DHT‐treated mice (*n* = 10). (E) Immunohistochemistry for TF protein on anterior prostates of sham‐operated, castrated and castrated DHT‐treated mice (*n* = 3). (F) Immunohistochemistry for EGR1 protein on anterior prostates of sham‐operated, castrated and castrated DHT‐treated mice (*n* = 3). (G) qPCR for IκBα mRNA in anterior prostates of sham‐operated, castrated, sham‐operated DHT‐treated and castrated DHT‐treated mice (*n* = 10). (H) qPCR for specificity protein 1 (SP1) mRNA in anterior prostates of sham‐operated, castrated, sham‐operated DHT‐treated and castrated DHT‐treated mice (*n* = 10). ***P ≤ 0.001, ****P ≤ 0.0001. Error bars represent standard error of the mean. [Color figure can be viewed at wileyonlinelibrary.com]

To address whether AR‐mediated regulation of TF is also relevant *in vivo*, we castrated or sham‐operated 8–10‐week‐old male C57BL6/J mice, and determined TF mRNA and protein expression in anterior and dorsal prostates. In addition, we included DHT‐treated sham‐operated and castrated mice as controls in our analysis. We observed that TF mRNA expression was significantly upregulated in anterior and dorsal prostates of castrated mice (Fig. 5A,C). This upregulation was significantly reduced after addition of DHT. A similar pattern was observed for EGR1 mRNA expression (Fig. 5B,D). Next, we performed immunohistochemical staining for TF and EGR1 proteins on anterior prostates of castrated mice. We observed upregulation of TF and EGR1 protein expression in epithelial cells of anterior prostates (Fig. 5E,F). Next, we wanted to determine whether castration also upregulated NF‐κB activity, which might contribute to the observed upregulation of TF in prostate epithelial cells. Indeed, we observed elevated IκBα mRNA expression levels in anterior prostates of castrated mice (Fig. 5G), suggesting that the NF‐κB signaling pathway is activated by castration. SP1 mRNA expression levels were not significantly altered in anterior prostates among all groups of mice tested (Fig. 5H). In summary, these data indicate that castration leads to upregulation of TF expression in prostate epithelial cells *in vivo*. As a final step, we wanted to verify whether an AR–EGR1–NF‐κB–TF signaling axis is also active in prostate cancer patients. Therefore, we used GSEA based on a published list of AR‐repressed and AR‐induced genes 24. Principally, we determined whether TF, EGR1, SP1, p65, p50, p52, IκBα, SP1 or c‐Rel gene expression was enriched among AR‐repressed or AR‐induced genes in a published microarray dataset 25. In line with an AR‐repressive function on TF, EGR1 and IκBα genes, we observed enrichment of the expression profile of these genes among AR‐repressed genes (Fig. S1), but not of the other genes tested. We conclude that TF, NF‐κB and EGR1 expression may be repressed by AR in prostate cancer patients, and that this signaling axis could also be active in human prostate epithelial cells *in vivo*.

## Discussion

We show here that TF expression is mediated through AR *in vitro*. Furthermore, castration leads to upregulation of TF expression *in vivo*. *In vitro*, in the AR‐dependent cell line LNCaP, NF‐κB and EGR1 mediate DHT‐induced TF repression. NF‐κB appears to be important for basal TF expression in prostate cancer epithelial cells.

In line with a regulatory function for AR *in vivo*, we observed elevated TF expression levels in prostate epithelial cells of castrated mice. Furthermore, castration upregulated EGR1 expression. These findings are in line with the *in vitro* findings, and suggest that TF is upregulated by EGR1 *in vivo*. In general, our combined results point to castration‐mediated induction of TF expression in prostate epithelial cells.

EGR1 is an important factor in prostate cancer, as it was shown to be overexpressed in malignant prostate cancer tissues 29, and EGR1‐deficient mice show impaired prostate tumorigenesis 30. In addition, EGR1 expression is elevated in androgen‐deprived LNCaP cells, and could have a role in the progression to castration‐resistant prostate cancer after prolonged ADT 31. Our data lend further support for an important role for EGR1, and establish EGR1 as an important upstream regulator of TF expression in an androgen‐deprived setting. Furthermore, it was previously reported that PTEN deficiency in mice leads to a similar transcriptional output in the prostate as castration in wild‐type mice. In line with our observations on castrated mice, PTEN loss enhanced the expression of EGR1 24. Analogous to castration, PTEN deficiency could thus also enhance TF expression by upregulating EGR1. However, further experiments in PTEN‐deficient mice are warranted to validate TF upregulation by PTEN deficiency.

In addition, we identify the NF‐κB signaling pathway as an important regulator for AR‐mediated TF regulation. A mutual negative regulatory function for the AR and the NF‐κB signaling pathway has been previously described by others 32, 33. Our results are in line with these observations, and further illustrate the importance of this negative regulatory loop. In addition, there is accumulating evidence that NF‐κB is involved in prostate cancer progression. In particular, it was previously shown that activation of NF‐κB is associated with biochemical relapse and a shorter time to disease recurrence 34, 35, 36. It is thus possible that TF mediates some of these NF‐κB functions as a NF‐κB downstream target. However, further research is needed to determine whether TF is an NF‐κB mediator during prostate cancer progression.

Overall, we believe, that our findings are especially important in the context of ADT, as it was previously shown that the VTE rate is increased in androgen‐deprived prostate cancer patients 6, 7. It is thus likely that, in prostate cancer patients, castration leads to upregulation of NF‐κB, EGR1 and TF expression in prostate epithelial cells. This upregulation of TF activity in prostate epithelial cells could contribute to increased MV TF activity in the blood of androgen‐deprived patients. However, it is important to state that ADT does not usually decrease the testosterone concentration to zero in prostate cancer patients. The currently accepted castration threshold for prostate cancer patients is 50 ng dL^−1^ (∼1.7 nm) 37. It is currently unclear whether this concentration is sufficient to maintain AR‐mediated TF repression, and further clinical studies are warranted.

Taken together, our data indicate a novel, crucial role for AR in reducing TF expression, which could be important for increased TF expression and TF‐positive MV release in androgen‐deprived prostate cancer patients, and contribute to elevated VTE rates in prostate cancer patients.

## Addendum

B. Hoesel conceptualized and coordinated the project, and designed experiments. B. Hoesel, A. Assinger, N. Mackman, and J. Schmid interpreted data and wrote the manuscript. B. Hoesel, M. Mussbacher, B. Dikorman, M. Salzmann, A. Assinger, B. Moser, and H. Paar performed experiments. L. Hell, J. Thaler, and U. Resch provided research tools. J. Basílio performed bioinformatics. All authors approved the final version of the manuscript.

## Disclosure of Conflict of Interests

The authors state that they have no conflict of interest.

## Supporting information


**Table S1.** Primer sequences used in this study.
**Fig. S1.** Gene set enrichment analysis of a published microarray dataset (GSE21032). TF, EGR1 and IκBα show an AR‐repressed gene signature.
